# The Impact of Climate on the Energetics of Overwintering Paper Wasp Gynes (*Polistes dominula* and *Polistes gallicus*)

**DOI:** 10.3390/insects14110849

**Published:** 2023-10-31

**Authors:** Helmut Kovac, Helmut Käfer, Iacopo Petrocelli, Astrid B. Amstrup, Anton Stabentheiner

**Affiliations:** 1 Institute of Biology, University of Graz, Universitätsplatz 2, 8010 Graz, Austria; 2 Dipartimento di Biologia, Università di Firenze, Via Madonna del Piano 6, 50019 Sesto Fiorentino, Italy; 3 Department of Biology—Genetics, Ecology and Evolution, 8000 Aarhus, Denmark

**Keywords:** *Polistes dominula*, *Polistes gallicus*, gynes, climate, energetics, overwintering

## Abstract

**Simple Summary:**

During overwintering diapause, the gynes of paper wasps (*Polistes* sp.) are mainly dormant in sheltered hibernacles, protecting them against predators and adverse weather conditions but hardly against low temperatures. By measuring the temperature inside hibernacles occupied by species from both Mediterranean (Italian; *P. dominula*, *P. gallicus*) and temperate (Austrian; *P. dominula*) climates (mean hibernacle temperatures: 8.5 °C and 3.2 °C, respectively), we were able to calculate the energetic demand of overwintering. The cumulative energetic costs differed between the populations. Costs were lowest for the *P. dominula* population from the cooler Austrian winter climate and significantly higher in *P. dominula* and *P. gallicus* from the warmer Italian climate. The lower costs of the temperate species were a result of the lower winter temperature and physiological acclimation processes. Energetic calculations with an assumed temperature increase of up to 3 °C due to climate change predict a dramatic increase of up to 40% in overwintering costs in all species.

**Abstract:**

Gynes of paper wasps (*Polistes* sp.) spend the cold season in sheltered hibernacles. These hibernacles protect against predators and adverse weather conditions but offer only limited protection against low temperatures. During overwintering diapause, wasps live on the energy they store. We investigated the hibernacles’ microclimate conditions of species from the Mediterranean (Italy, *P. dominula*, *P. gallicus*) and temperate (Austria, *P. dominula*) climates in order to describe the environmental conditions and calculate the energetic demand of overwintering according to standard metabolic rate functions. The temperatures at the hibernacles differed significantly between the Mediterranean and temperate habitats (average in Austria: 3.2 ± 5.71 °C, in Italy: 8.5 ± 5.29 °C). In both habitats, the hibernacle temperatures showed variance, but the mean hibernacle temperature corresponded closely to the meteorological climate data. Cumulative mass-specific energetic costs over the studied period were the lowest for the temperate *P. dominula* population compared with both Mediterranean species. The lower costs of the temperate species were a result of the lower hibernacle temperature and acclimation to lower environmental temperatures. Model calculations with an increased mean temperature of up to 3 °C due to climate change indicate a dramatic increase of up to 40% in additional costs.

## 1. Introduction

Winter in climate regions with seasonal changes is a challenge for overwintering insects, forcing them to adapt their behaviour and physiology to cope with unfavourable environmental conditions. The gynes of paper wasps (*Polistes* sp.) spend the cold season in sheltered retreats called winter hibernacles. While these hibernacles offer protection against predators and various environmental challenges, such as rain and snowfall, they provide limited defence against low ambient temperatures during winter (our own observation). Exposure to low-temperature extremes can directly cause mortality by freezing or lead to water deficits and energy depletion, threatening the wasps’ survival. To cope with these harmful conditions, overwintering wasps, like many other insect species, spend the winter in a state of dormancy (diapause or quiescence [1]), in which activity is low, and the metabolism is suppressed [2,3,4]. Feeding and the synthesis of energy stores cease before entering the dormant phase, and they live on their reserves until spring. Energy stores must be sufficient to fuel the mechanisms and physiological processes that protect against cold and desiccation. Additionally, they have to drive the basal metabolism throughout the winter and early spring, when prey is scarce, and foraging conditions are poor. Paper wasps start foraging only on warm days when outside temperatures rise above ~18–20 °C (personal observation).

During overwintering, wasps are mostly dormant and ectothermic, and therefore their metabolic rate is exponentially related to ambient temperature [3,4,5,6]. The exponential nature of the metabolic rate–temperature relationship means that increases in temperature will have a great impact on overwintering energy expenditure. As a consequence of this relation, the remaining energy reserves of wasps in spring are directly determined by the climate and the thermal conditions in the hibernacles (microclimate). This means that the thermal environment conditions in the microhabitat (hibernacles) are of crucial importance for survival and fitness. Specifically, thermal variability could be particularly important in determining the overwintering energy expenditure due to the effect of Jensen’s inequality [7]. Jensen’s inequality describes how an accelerating metabolic rate–temperature relationship leads to a higher mean metabolic rate and, consequently, increased energy consumption. This occurs because, in exponential functions, the sections in the higher range of thermal viability cause a higher increase in metabolic rate than the reductions in energy demand in the lower temperature range [7,8]. Thus, a great part of the cumulative energy expenditure during overwintering can be accounted for by (diurnal) thermal fluctuations to higher temperatures, in addition to a warm (diurnal) mean (e.g., during autumn, when wasps are already in the hibernacles, or in spring when they still remain in it) [9,10]. Because of the disproportionate increase in metabolism with temperature, one can argue that small changes in the microhabitat conditions could have a big influence on overwinter energy drain. Warmer overwintering temperatures lead to higher rates of energy consumption and a concurrent depletion of stored reserves. The increased resource consumption could have a negative impact not only on survival but also on fecundity, as less energy resources are available for reproduction in the spring (e.g., [7,11,12,13,14,15,16,17,18,19,20,21,22]).

Anthropogenic climate change is altering annual mean temperatures as well as winter temperatures [23]. As already mentioned above, energetics can be affected by changes in the mean temperature, increased thermal variability, and more frequent thermal extremes. Current climate change prediction models do a poor job of predicting extreme temperatures and temperature variability, and the scale of climate documentation is unsuitable when examining insects in their microhabitats [24]. To identify the ecological consequences of climate change on the animal world, macroecological approaches and climatological tools (e.g., WorldClim climate data provider, https://worldclim.org/ (accessed on 20 December 2022)) have been developed. These tools and approaches can be used in predictive models to anticipate the consequences of climate warming and future changes in biological systems. Global temperature models, however, often ignore fine-scale microhabitat deviations. More accurate calculations can be achieved by using the actual microclimatic conditions experienced by organisms in their habitat [24,25,26,27,28,29,30,31]. In breeding (summer) colonies of *Polistes* wasps, considerable deviations of microhabitat temperatures from standard meteorological temperatures were observed [32]. It has remained unclear, however, whether this is also the case in wasps’ overwintering hibernacles. Therefore, we investigated the environmental conditions at the overwintering sites of paper wasps in the temperate climate of Austria (Styria, *P. dominula*) and the Mediterranean climate of Italy (Tuscany; *P. dominula*, *P. gallicus*). Our objective was the characterisation and comparison of microclimates from different habitats and climate regions, illustrating the consequences for the energetics during overwintering. Based on these measurements, we modelled the energetic demand of wasps for overwintering according to the known metabolic rate–temperature relationships of overwintering gynes [3]. The results revealed the differences in energetic costs of the two species and among different populations, attributable to distinct environmental conditions in their habitats and their respective metabolic adaptations. The simulation of future climate conditions with increasing temperatures revealed possible increases in the costs of overwintering.

## 2. Materials and Methods

### 2.1. Species, Locations, and Climates

**Figure 1:**
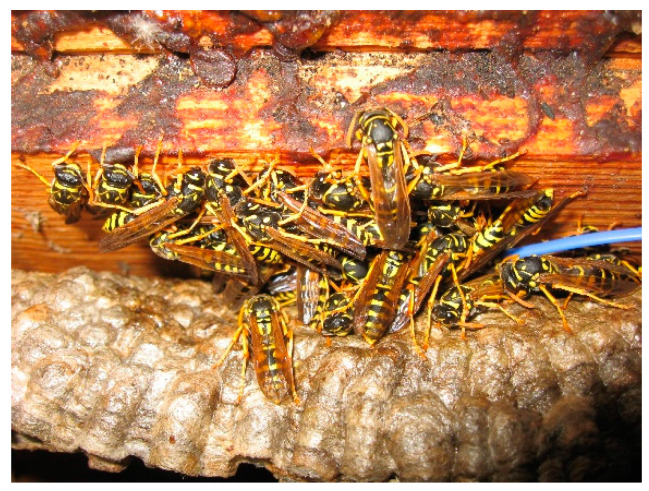
Hibernacle of paper wasp gynes of *Polistes dominula* in Austria near an old nest.

The investigations were conducted on seven hibernacles of *Polistes dominula* in Styria (Austria, AT, central Europe, temperate climate) and on seven “mixed” hibernacles of *Polistes dominula* and *Polistes gallicus* in Tuscany (Italy, IT, southern Europe, Mediterranean climate). In Austria, the wasps had their hibernacles in wooden bird boxes or empty bee hives, often in the same place they had nested (Figure 1). In Italy, we found the wasps’ hibernacles under the bark of grapevine, in a shed, and in grave lanterns at a cemetery. In Italy, it is very common that the two species, *Polistes dominula* and *Polistes gallicus*, are found mixed in the same hibernacle [33] (personal observation).

**Figure 2:**
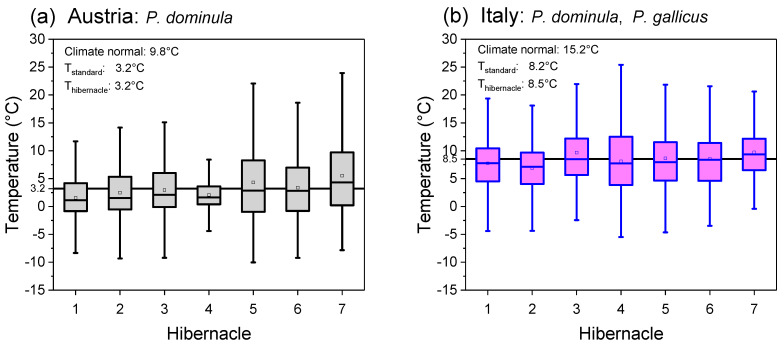
Ambient air temperatures at paper wasp hibernacles during overwintering (December–March) in (**a**) Austria and (**b**) Italy. Box and whisker plots represent median temperatures with first and third quartiles; dots in plots are means. The solid black line in the background indicates the mean of all nests. The mean meteorological standard ambient air temperature (December–March 2017–2020) and the climate normal values (1981–2010) of the nearest weather stations in Austria (Graz, ~20 km distance) and Italy (Florence, ~10–20 km) are indicated.

To describe the different climates of the two habitats, the meteorological climate normal values (Austria: ZAMG, Klimamittelwerte für den Zeitraum 1981–2010 [34]; Italy: LaMMA Consorzio, Climatologia di Firenze 1981–2010 [35]) of the nearest weather stations in Austria (Graz) and Italy (Florence) were evaluated, indicated as “Climate normal” in Figure 2. Furthermore, for comparison with our microclimate measurements, the means of the meteorological standard air temperature measurement (T_standard_, 2 m in height and shaded) of these weather stations (Graz and Florence) were calculated for the measurement period (December–March 2017–2020).

### 2.2. Temperature Measurement

The measurements were conducted during the time the wasps spent permanently in their overwintering hibernacles (December to March, in the years 2017–2020). The ambient air temperature was continuously recorded with thermistor probes or thermocouples positioned 2–4 cm from the wasps. The temperature sensors were connected to data loggers (MSR Electronics GmbH, Seuzach, Switzerland; or Extech SD 200, FLIR Commercial Systems, Nashua, NH, USA), which recorded the data in 10 min intervals.

### 2.3. Energetic Expenditure Calculations

**Figure 3:**
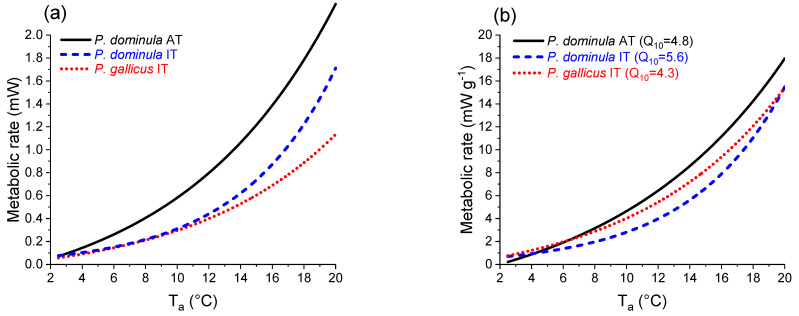
(**a**) Individual and (**b**) mass-specific standard metabolic rate of paper wasp gynes from Austria (*P. dominula* AT) and Italy (*P. dominula* IT and *P. gallicus* IT) in relation to ambient air temperature (T_a_), modified from Kovac et al. [3]. The Q_10_ values (quotient of T_a_: 7.5 °C to 17.5 °C) of the metabolic functions are indicated; fit functions, parameters, and statistical details are provided in Table S1.

Energetic expenditure calculations were conducted by using models describing the relationship between temperature and metabolic rate from a previous study [3,32] (Figure 3, Table S1). The model is based on respiration rates measured in the two species we investigated in the present study. The origin of the species and the habitats in that study were the same as those in the present study.

For each wasp species and hibernacle, the individuals’ carbon dioxide production was calculated at 10 min intervals (which was the interval of temperature recordings) by using the temperature measurements of this study and the equations of the standard metabolic rate (SMR: resting metabolic rate) from Kovac et al. [3]. The standard metabolic rate was chosen as it was measured in resting wasps and represents the metabolic costs of basal subsistence, which mainly accounts for the energetic demand during overwintering. 

Additionally, we conducted the same calculations using the means of the meteorological standardised air temperature measurements (T_standard_, December to March 2017–2020, 2 m in height and shaded) of the nearest meteorological weather stations (Graz: 3.2 °C, Florence: 7.0 °C). We also simulated additional energetic costs for increased temperatures due to climate change, according to an increase of 1, 2, and 3 °C of the mean hibernacle temperature.

The wasps’ energy turnover (J s^−1^ g^−1^) was calculated using the respiratory metabolic fit functions from Kovac et al. [3] (Figure 3) and the measured hibernacle temperatures. For this purpose, we first transformed the CO_2_ production with the respiratory quotient determined for each species (RQ, *P. dominula* AT: 0.78; *P. dominula* IT: 0.80; *P. gallicus* IT: 0.78 [3]) to O_2_ consumption and then multiplied the O_2_ consumption by the adequate caloric equivalent (RQ = 0.78: 20.10 kJ L^−1^ O_2_; RQ = 0.80: 20.19 kJ L^−1^ O_2_; see Silbernagel and Despopoulos (1991) [22]). Then, the energetic turnover was calculated chronologically for the ten-minute intervals and summed up for the entire investigation period (cumulative costs from December to March, 121 days). Since the species differed significantly in weight, we used the mass-specific metabolic rate for calculations and statistics. We should note that these calculations represent only the energetic costs of resting individuals. However, we presume that the wasps are mainly quiescent during overwintering and that this model represents a reliable estimation of the main energetic costs of overwintering.

### 2.4. Data Analysis and Statistics

The calculations were performed with MS Excel software (version 2108, LTSC MSO (16.0.14332.20579); Microsoft Corporation, Redmond, WA, USA), and curve plotting was carried out using Origin 2017G software (version 94G, b 9.4.0.220; OriginLab Corporation, Northampton, MA, USA). The statistics were determined with Statgraphics software (version 18.1.01; Statgraphics Centurion XVI, StatPoint Technology Inc., The Plains, VA, USA). Nonparametric Mann–Whitney or Kruskal–Wallis tests were used to compare the hibernacles’ temperatures, the temperature data of the climatic regions, and the metabolic data of the species, and the Bonferroni test was used for the pairwise comparison of these data.

## 3. Results

### 3.1. Hibernacle Micro- and Macroclimate

In both study habitats, hibernacle temperatures varied between locations. Nevertheless, the mean hibernacle temperatures revealed a significant difference in the hibernacle microclimate between the habitats in Austria and Italy during the overwintering period (T_hibernacle_, December–March 2017–2020; *p* = 0.0022, Mann–Whitney test). The mean ambient air temperature (±SD) in the seven *P. dominula* hibernacles in Austria was 3.2 ± 5.71 °C. In the seven mixed hibernacles of *P. dominula* and *P. gallicus* in Italy, we measured 8.5 ± 5.29 °C on average (Figure 2).

The meteorological standard ambient temperature means (T_standard_, December–March, in the years 2017–2020) recorded at the nearest weather stations also differed significantly (*p* < 0.0001, Mann–Whitney test). The temperature was determined to be 3.2 ± 5.02 °C for Graz (Austria) and 8.2 ± 4.43 °C for Florence (Italy). In both habitats, the seasonal mean of hibernacle temperature was almost the same as the meteorological standard temperature recorded by the nearest weather stations (difference Austria: −0.02 °C, Italy: +0.24 °C).

The meteorological climate normal values of the period 1981–2010 show the difference between the temperate climate of central Europe and the southern Mediterranean climate of Italy. The climate normal temperature of the nearest weather station in Austria (Graz) was 9.8 °C, and in Italy (Florence), it was 15.2 °C (Austria: ZAMG, Klimamittelwerte für den Zeitraum 1981–2010 [34]; Italy: LaMMA Consorzio, Climatologia di Firenze 1981–2010 [35]). 

### 3.2. Energetic Costs of Overwintering

**Figure 4:**
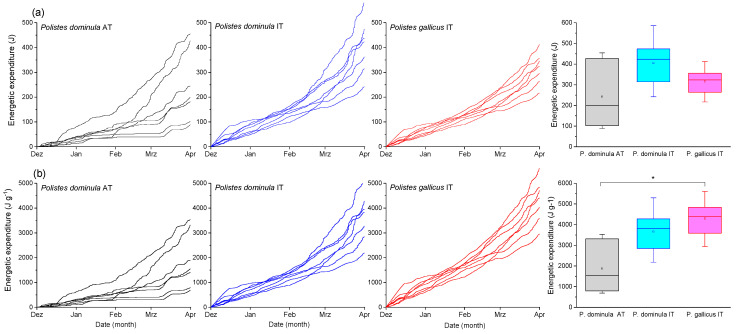
(**a**) Individual and (**b**) mass-specific energetic expenditure of paper wasp gynes from Austria (*P. dominula* AT) and Italy (*P. dominula* IT and *P. gallicus* IT) summed up for the overwintering period (December–March 2017–2020). Data were calculated from ambient air temperature in the hibernacles (T_hibernacle_) and the metabolic rate–temperature model from Kovac et al. [3]. Box and whisker plots represent median temperatures with first and third quartiles; dots in plots are means. Significance: * *p* < 0.05.

**Figure 5:**
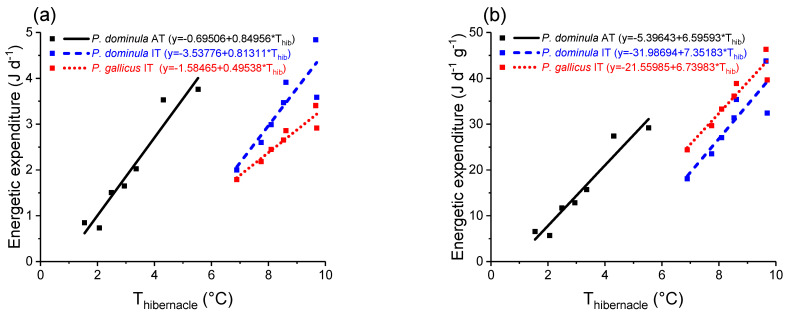
(**a**) Individual and (**b**) mass-specific daily energetic expenditure of paper wasp gynes from Austria (*P. dominula* AT) and Italy (*P. dominula* IT and *P. gallicus* IT) in relation to mean hibernacle temperature (T_hibernacle_).

The wasps’ energetic expenditure was calculated with the standard metabolic rate model established by Kovac et al. [3] (SMR model, Figure 3) for the resting insects for their entire overwintering period (December–March). The mass-specific standard metabolic rate of *P. dominula* from Austria differed significantly from *P. dominula* IT (*p* < 0.0001, ANOVA) and *P. gallicus* IT (*p* < 0.05, ANOVA). Calculations were performed with hibernacle air temperature as the independent variable and energy turnover derived from metabolic data (Figure 3, W g^−1^) as the dependent variable. The results revealed no significant differences for the summed-up energetic values for the SMR during the observation period without considering the wasps’ differing mass (*p* > 0.05, Kruskal–Wallis test; Figure 4a). However, the wasps’ mass-specific metabolic rate revealed significant differences for the summed-up energetic values (cumulative costs from December–March, *p* < 0.01, Kruskal–Wallis test; Figure 4b). While *P. dominula* from Austria had significantly lower energetic costs than *P. gallicus* from Italy (*p* < 0.05, Bonferroni test), they were not significantly different from *P. dominula* from Italy (*p* > 0.05, Bonferroni test). The mass-specific energetic costs were 1883.6 J g^−1^ in *P. dominula* AT, 3664.4 J g^−1^ in *P. dominula* IT, and 4301.3 J g^−1^ in *P. gallicus* IT for the observation period December–March (Figure 4b). The daily costs (J d^−1^ g^−1^) increased linearly with ambient temperature in all species and habitats (Figure 5). *P. dominula* from Austria had significantly lower daily energetic costs than both Italian species (difference in intercept *p* < 0.002, ANOVA). The two Italian species differed significantly from each other; *P. gallicus* IT had significantly higher costs than *P. dominula* IT (difference in intercept *p* < 0.02, ANOVA).

**Figure 6:**
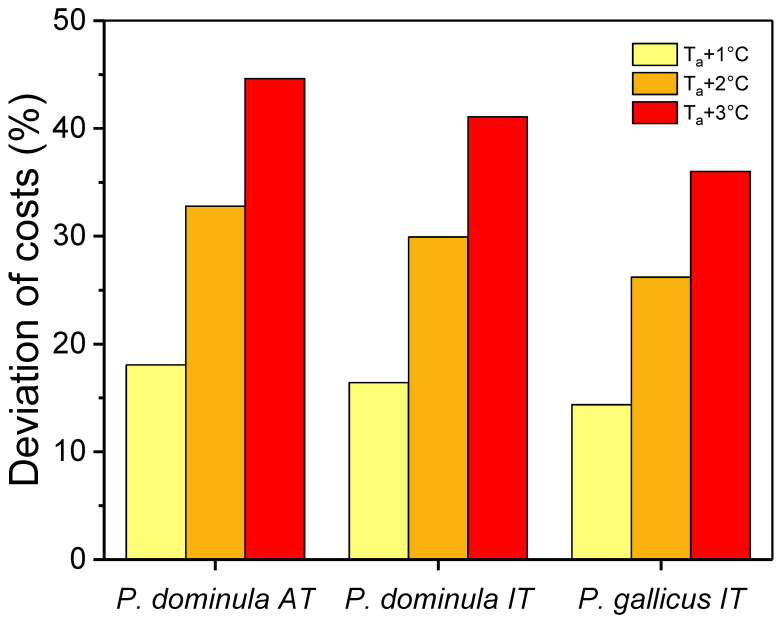
Percent deviation in energetic costs of paper wasp gynes during overwintering in future climate scenarios, with a temperature increase of 1, 2, and 3 °C above ambient air temperature in comparison to our microclimate measurements (T_hibernacle_) from December to March of 2017–2020. Calculations were performed for hibernacle temperatures in Austria (*P. dominula* AT) and Italy (*P. dominula* IT and *P. gallicus* IT).

The simulation of an increased ambient temperature of 1, 2, and 3 °C resulted in drastically higher energetic costs. Calculated cost increases amounted to 18.1%, 32.8%, and 44.6% for *P. dominula* AT; 16.4%, 29.9%, and 41.1% for *P. dominula* IT; and 14.4%, 26.2%, and 36.0% for *P. gallicus* IT, respectively (Figure 6).

## 4. Discussion

The calculation of the energy expenditure of overwintering paper wasp gynes allows for the comparison of how two different climates affect the overwintering energy expenditure. The prerequisite of such estimations, however, is the accurate knowledge of thermal conditions in winter hibernacles. Temperature measurements in the microhabitats of wasps from different climates yielded an expected result; hibernacle temperatures during overwintering in the Mediterranean climate of Italy were higher than those in the temperate climate of Austria (Figure 2). Rather surprisingly, however, the average of mean hibernacle temperatures was quite similar to the standard meteorological temperature data of the nearest weather stations, ~20 km away in Austria and ~10–20 km away in Italy (Figure 2). The thermal microhabitat conditions in the hibernacles chosen by paper wasps are much more diverse (Figure 5). Nevertheless, we assume that the wasps were experiencing conditions similar to the air temperature outside the hibernacles. We presume that wasps do not actively search for hibernacles with more favourable (warmer) microclimate conditions. Instead, the hibernacle should simply protect from harsh weather conditions (rain, hail, and snow) and predators. A similar behaviour in the choice of winter hibernacles was observed by Gibo [36] in *Polistes fuscatus* in Toronto, Canada. 

It is well known and documented that many wasp gynes do not survive overwintering [37,38,39,40]. The question arises as to what the consequences of (low) temperature are, and in which way the microclimate determines the overwintering success of wasps. If we consider winter, one would expect that lower temperatures mean much worse conditions for survival. However, this is only valid for very low temperatures in the range of an insect’s supercooling point. The supercooling point of paper wasps is not known, but Barnes et al. [41] reported a supercooling point for *Vespula vulgaris* queens of −16.9 °C. Gibo [37] showed that 90% of paper wasp queens (*Polistes fuscatus*) survived exposure to −15 °C, but no wasp survived when exposed to −20 °C for 48 h. In another study [36], a few wasps survived even −20 °C after 48 h exposure, but none were able to survive −25 °C. These results are in good accordance with our own measurements on body and air temperature in a *Polistes dominula* hibernacle (in preparation). Down to a temperature of about −15 °C, all of the 24 wasps were still alive and showed no signs of damage. All but one had left the hibernacle in spring.

In the context of metabolism and energetic expenditure of ectothermic insects, the situation is complex. Temperature directly determines the rates and pathways of metabolic processes (e.g., [4,42]). A higher temperature increases whole-animal metabolism and thus inevitably the consumption of stored energy resources of the insects. Sgolastra et al. [9,43] showed that warmer winter temperatures accelerate the diapause development in *Osmia lignaria*, which results in a reduction in the hibernation period. This is accomplished at high metabolic costs in terms of weight loss and fat body depletion, resulting in increased winter mortality. 

Like in many other insects, the metabolism of paper wasp gynes increases disproportionately with temperature. The exponential course is especially pronounced at higher temperatures above 10 °C [3] (Figure 3). Our model considers the change in the metabolism rate–temperature relationship when the wasps acclimate to winter conditions. Overwintering wasps have to live on the reserves that they have accumulated in autumn, and they still need reserves in spring to build a new nest and overcome periods of bad weather. An energy-saving overwintering strategy is therefore absolutely necessary. In overwintering insects, this can be achieved by suppressing metabolism [2,8,44,45]. This strategy was also observed in overwintering paper wasp gynes, which had a lower metabolic rate than summer individuals [3]. These investigations show that populations of the same species (*P. dominula*) as well as of a closely related sister species (*P. gallicus*) from differing climates differ in their metabolic response (sensitivity) to temperature (see Figure 3). Paper wasps from the cooler, temperate Austrian climate (*P. dominula* AT) had a higher mass-specific standard metabolic rate than those from the warmer Mediterranean climate in Italy (*P. dominula* IT, *P. gallicus* IT). This adaptation is presumed to be a response to environmental (temperature) variation. In some other insect species, it has been shown that the metabolic rate response to temperature can differ among populations and between species [46,47,48,49]. This means that during overwintering, wasps from the Mediterranean climate exhibit a reduced metabolism in comparison to wasps from the temperate climate in order to compensate for the higher energetic demand due to higher ambient temperatures. Our model calculations of the summed-up energy expenditure for the overwintering period (cumulative costs from December to March, Figure 4) show that, in the warmer (Italian) habitat, mass-specific energetic costs are higher (Figure 4b), although the Italian species partially compensate for the effects of higher ambient temperatures by a somewhat lower winter metabolic rate (Figure 3). 

As already mentioned, the metabolic rate increases exponentially with temperature [3] (Figure 3), leading to a disproportionally higher energetic demand during the warm parts of the day. The calculated mean daily costs increase linearly with the hibernacle temperature in all species (Figure 5). The calculation of the daily energetic demand clearly shows that the choice of a certain overwintering site, and thus microclimate, may have a considerable effect on energetic costs. In *P. dominula* AT, the maximum difference of ~5 °C in the mean hibernacle temperature resulted in a difference in mass-specific energy expenditure by a factor of about 6.5, and in both *P. dominula* IT and *P. gallicus* IT, a difference of ~3 °C resulted in a span of energy expenditure by a factor of about 2 (Figure 5b). This strong influence of microclimate on the energetics is similar to summer individuals (workers) of paper wasps: cumulative energy costs during a breeding season are highest in *P. gallicus* from the warm Mediterranean climate and lowest in *P. biglumis* from the harsh Alpine climate [32].

Metabolic measurement is an appropriate tool to assess a species’ vulnerability to climate warming, and the energy supply (amount of energy stores) is essential in this context. As the microhabitats investigated in this study were obviously not buffered from the impact of ambient temperature, we simulated the energetic costs under future climate conditions with the simple assumption of a 1 to 3 °C elevated ambient air temperature during overwintering (Figure 6) (see also [50]). Our calculations suggest that the energetic costs will increase in a similar course in all species: up to about 36% additional costs in *P. gallicus* IT, 41% in *P. dominula* IT, and 45% in *P. dominula* AT under a 3 °C increase scenario. This assumption seems quite realistic if one considers that the 1.5 °C increase in temperature due to climate change has already been reached on land. This simple assumption, however, only considers a mean increase in ambient temperature. Climate change may lead to increases in thermal variability, and range expansion or dispersion to new habitats could shift species to more variable environments. The predicted increases in mean temperatures will shift daily thermal cycles to fluctuate at a point where the relationship of metabolic rate to temperature is steeper, and this will enhance energetic drain [51]. If there are extraordinarily higher temperatures in autumn or spring, when overall temperatures are already relatively high, the metabolism and energetic costs will increase disproportionately due to the exponential metabolic rate–temperature relationship (Figure 3) and will potentially have a significant impact on overwintering energetics. We expect these changes to negatively impact overwintering success in ectotherms that lack the ability to reduce the thermal sensitivity of their winter metabolism. To answer the question of whether any species of paper wasps (and if yes, which ones) are threatened by extinction or range shifts in future climate scenarios, we will have to determine the energy resources wasps have accumulated in autumn and the remaining stored energy reserves in spring.

## Data Availability

All data are available in the manuscript or the Supplementary Materials.

## Supplementary Materials

The following supporting information can be downloaded at: https://www.mdpi.com/article/10.3390/insects14110849/s1, Table S1: Statistical details and fit parameters.
