# Supplementary material for: The Impact of Climate on the Energetics of Overwintering Paper Wasp Gynes (Polistes dominula and Polistes gallicus)
Source: Insects. 2023 Oct 31;14(11):849. doi: 10.3390/insects14110849 (PMC10672273; doi:10.3390/insects14110849)
Supplement: Supplementary file 1 [file insects-14-00849-s001.zip › insects-2647031-supplementary.pdf]

insects

MDPI

Article

Energetics of overwintering paper wasp gynes from differing climates (*Polistes dominula* and *Polistes gallicus*).

Helmut Kovac[1*], Helmut Käfer[1], Iacopo Petrocelli[2], Astrid B. Amstrup[1,3], Anton Stabentheiner[1*]

[1] Institute of Biology, University of Graz, Universitätsplatz 2, 8010 Graz, Austria
[2] Dipartimento di Biologia, Università di Firenze, Via Madonna del Piano, 6 – 50019 Sesto Fiorentino, Italy
[3] Department of Biology - Genetics, Ecology and Evolution, 8000 Aarhus C, Denmark
* Correspondence: helmut.kovac@uni-graz.at, anton.stabentheiner@uni-graz.at

Supplementary information

Table S1**.** Statistical details and the fit parameters of fit functions in Figure 3 of the individual and the mass specific standard metabolic rate (SMR=y0 + A*exp(R0*T_a)) of paper wasp gynes from Austria (*P. dominula* AT) and Italy (*P. dominula* IT, *P. gallicus* IT), data from Kovac et al. [32].

Individual metabolic rate

Species	Parameter			R^2	P	N
	y0	A	R0			
***P. dominula* AT**	-0.55425	0.4633	0.09114	0.51872	<0.0001	929
***P. dominula* IT**	-0.01822	0.06261	0.16594	0.78001	<0.0001	674
***P. gallicus* IT**	-0.13431	0.1457	0.1082	0.70700	<0.0001	1019

Mass specific metabolic rate

Species	Parameter			R^2	P	N
	y0	A	R0			
***P. dominula* AT**	-0.43032	0.35971	0.09114	0.51872	<0.0001	929
***P. dominula* IT**	-0.01648	0.05661	0.16594	0.78001	<0.0001	674
***P. gallicus* IT**	-0.18273	0.19824	0.10820	0.70700	<0.0001	1019
